# Ethical implications of screening for loneliness in primary care when intervention capacity is limited

**DOI:** 10.1186/s12910-026-01568-3

**Published:** 2026-08-11

**Authors:** Alison Warren, Patrick G. Corr

**Affiliations:** 1 George Washington University, School of Medicine and Health Sciences, 2600 Virginia Ave NW, Suite 300, Washington, DC 20037 United States; 2 Division of Continuing Education, Harvard University Extension School, Cambridge, United States

**Keywords:** Loneliness screening, Medical ethics, Social determinants of health, Screening ethics

## Abstract

**Background:**

Loneliness has emerged as a clinically significant health concern, prompting growing interest in systematic screening in care settings. However, the ethical justification for such screening remains uncertain when intervention capacity is limited or inequitably distributed.

**Main body:**

Drawing on Wilson and Jungner’s classical screening criteria and contemporary social risk screening literature, we argue that screening without response capacity violates core ethical principles including autonomy, beneficence, nonmaleficence, and justice. Potential ethical tensions are not hypothetical but are documented across implementation studies and qualitative research on social needs screening.

**Conclusion:**

Ethically defensible loneliness screening requires alignment between identification and response capacity, including adequate referral pathways, clinician training, community partnerships, and transparent communication about the limits of available assistance.

## Background

Loneliness, a subjective and distressing perception of insufficient social connection, has emerged as an issue of increasing relevance in healthcare [1]. Loneliness is increasingly understood as a multidimensional phenomenon extending beyond the subjective perception of insufficient social connection [1, 2]. Contemporary conceptualizations emphasize that loneliness reflects discrepancies between desired and actual social relationships in terms of both quantity and quality, while also encompassing relational, psychological, cultural, and existential dimensions of human experience [3–6]. Persistent loneliness is subjective and emotionally painful [7] (Holt-Lundstad & Sine, 2025), and distinct from its more objective counterpart, social isolation, which constitutes an absence or deficiency of social interaction and may or may not give rise to distress [8–10]. As such, individuals may experience loneliness despite extensive social networks, while others who live alone or have relatively few social contacts may not experience loneliness [1, 10]. Because the ethical questions surrounding subjective disclosure, patient autonomy, and expectations of clinical response differ from those associated with objectively measured social isolation, the present analysis focuses specifically on loneliness while recognizing that future ethical work should consider integrated approaches to assessing both constructs [1, 11]. Screening or assessment for social isolation also warrants ethical consideration, particularly when objective indicators are used to characterize individuals who may not regard their circumstances as problematic. Integrated approaches may be clinically useful, but the concepts and corresponding responses should not be conflated.

Experiences of loneliness are shaped by developmental stage, life transitions, bereavement, social exclusion, structural disadvantage, and individual expectations regarding belonging and meaning [12–15]. Consequently, loneliness cannot be reduced to simple social contact deficits but represents a complex interaction between individual experience and broader social context [1, 2]. Epidemiologic evidence links loneliness to adverse health outcomes, including elevated morbidity and mortality risk, particularly among older adults [12, 16]. These associations have generated expanding interest in identifying loneliness within clinical settings, especially primary care, where patients with diverse medical, psychological, and social concerns routinely present [17]. Evidence from primary care populations further substantiates these concerns. Mullen and colleagues [11, 17] found that loneliness, measured using the three-item UCLA Loneliness Scale [18], was common among adult patients and associated with poorer self-rated health and greater health care utilization.

Interest in loneliness screening has emerged within broader efforts to integrate social and behavioral domains into health care delivery. Reviews and technical reports on social risk screening describe increasing institutional attention to identifying social factors within clinical workflows, often using brief standardized tools embedded in electronic health records [19, 20]. Loneliness screening shares features with other forms of social and psychosocial screening but presents distinct challenges. Unlike many biomedical conditions, loneliness is subjective, relational, context-dependent, and does not map cleanly onto standardized referral pathways. Yet this literature consistently notes substantial variability in the availability, effectiveness, and sustainability of interventions designed to address identified needs, as well as uncertainty regarding appropriate follow-up actions once needs are detected [20, 21].

These challenges are compounded by the realities of primary care practice, where visit time is constrained and clinicians must balance competing demands [22]. Although healthcare systems may identify loneliness during clinical encounters, loneliness is fundamentally a social, relational, and often existential experience that extends beyond the boundaries of medical care [1, 2]. Persistent loneliness reflects interactions among interpersonal relationships, life experiences, community contexts, and broader social and structural conditions rather than a discrete disease entity [5, 13, 23]. Clinical screening should therefore be understood as only one component of a broader societal response and should not be interpreted as implying that healthcare alone can resolve loneliness [12, 24]. Rather, its potential value lies in recognizing patients who may benefit from supportive conversations, care coordination, or connection with community resources when such resources are available [11, 24].

### Definition of the ethical issue

This paper examines the ethical implications of implementing loneliness screening in primary care when the capacity to respond meaningfully to identified loneliness is limited, variable, or inequitably distributed. The central ethical question is whether it is morally permissible to routinely screen for loneliness using brief tools such as the UCLA-3 in clinical settings where referral pathways, clinician time, training, and community-based resources are often insufficient to support effective follow-up [11, 25]. Classical screening ethics provides a relevant normative framework. Wilson and Jungner’s [26] (1968) foundational principles emphasize that screening is only justified when effective, supportive responses are available [26, 27]. Loneliness further complicates this ethical terrain because of challenges related to measurement and interpretation. Commentaries caution that loneliness is frequently conflated with living alone or social isolation, raising the risk of misclassification when brief screening tools are used without sufficient contextual understanding [1]. This creates concerns not only about *whether* screening should occur, but also about *how* screening results are interpreted, documented, and acted upon in routine care.

### Normative foundations: when is screening ethically justified?

Screening is not ethically neutral merely because it is intended to promote health. An organized screening program systematically seeks information from people who may not otherwise have presented with the condition or concern and may classify them as requiring further assessment or response. Ethical evaluation must therefore consider not only the validity of the screening instrument, but the entire pathway created by the program, including communication, interpretation, documentation, follow-up, access to support, and monitoring of benefits and harms. Wilson and Jungner’s 1968 principles provide a historically influential starting point for evaluating organized screening programs, although they do not constitute a comprehensive contemporary ethical framework. Their criteria address whether the condition represents an important health problem; whether its natural history is sufficiently understood; whether a suitable and acceptable test exists; whether facilities for further assessment and intervention are available; whether there is an agreed policy regarding whom to treat or support; whether the balance of costs and potential benefits is reasonable; and whether screening is implemented as a continuing program rather than as an isolated activity [26]. Wilson and Jungner placed particular emphasis on the availability of an effective response after detection, identifying adequate treatment capacity as among the most important prerequisites for screening.

Subsequent scholarship has shown, however, that these criteria should not be applied as a rigid checklist or transferred uncritically to every contemporary screening context. Dobrow and colleagues’ [28] (2018) consolidated principles shift attention from the isolated screening test to the screening program as a coordinated system and emphasize clear objectives, integration with health services, informed choice, equitable access, quality assurance, and ongoing evaluation of the full pathway. More recent reassessments similarly identify limitations in the original framework, including imprecise terminology, limited attention to program management, and insufficient incorporation of ethical, legal, societal, and stakeholder perspectives. Contemporary screening ethics therefore requires a multidimensional and learning-oriented approach capable of evaluating both intended benefits and unintended harms over time [28, 29].

The present analysis draws on this modernized programmatic interpretation rather than treating loneliness as a conventional screen-detectable disease. Loneliness lacks a single natural history, confirmatory diagnostic pathway, or standardized treatment threshold comparable to those assumed in many traditional screening programs. The relevance of the screening literature lies instead in its insistence that systematic identification should be assessed together with the processes that follow it. For loneliness, those processes may include contextual assessment, empathic acknowledgment, shared decision-making, brief counseling, care coordination, or connection with community-based resources rather than diagnosis and treatment in the conventional biomedical sense.

Importantly, advocating for ethical consideration of loneliness screening should not be interpreted as redefining loneliness as a disease. Loneliness is conceptualized here as a clinically relevant determinant of health that may warrant recognition when it contributes to distress, impaired functioning, or adverse health outcomes. Identification does not itself establish pathology, nor does a positive score determine that a patient requires a prescribed intervention. Importantly, advocating for ethical consideration of loneliness screening should not be interpreted as redefining loneliness as a disease [1, 12]. Rather, loneliness is conceptualized here as a clinically relevant determinant of health that may warrant recognition when it contributes to suffering, impaired functioning, or adverse health outcomes [15, 16, 30]. Similar to other psychosocial risk factors, identification does not imply pathology but instead may inform person-centered care when appropriate supports are available [1, 11]. Maintaining this distinction helps guard against medicalization while preserving the legitimate role of healthcare in recognizing and responding proportionately to social and relational suffering [1, 4, 19].

The clinical analysis that follows is informed by the four-principle approach to biomedical ethics outlined by Beauchamp & Childress in 1979 and now in its 8th edition (2019) [31]: autonomy, beneficence, nonmaleficence, and justice. These principles are used as a structured framework for examining patient choice, potential benefit, avoidable harm, professional responsibility, and the fair distribution of screening-related resources and outcomes.

### Ethical risks and harms of screening without response capacity

When loneliness screening is implemented without adequate capacity to respond, ethical risks emerge across multiple levels of the health care system. The four principles of biomedical ethics (autonomy, beneficence, nonmaleficence, and justice) provide a practical framework for analyzing these ethical questions while recognizing that no single principle is inherently paramount to ‘doing good medical ethics’ [32] and that their relative importance depends on the clinical context [31, 33]. These principles are used here as a structured clinical ethics framework rather than as a claim that each concern can be reduced to a single principle. In practice, the ethical implications of loneliness screening often involve overlapping questions concerning patient choice, potential benefit, avoidable harm, professional responsibility, and fair distribution of resources. 

### Patient-level ethical risks

From the perspective of autonomy, loneliness screening raises concerns related to autonomy, dignity, and the experience of disclosure. Loneliness is a subjective and potentially stigmatized experience, and patients may be reluctant to disclose social or relational distress in clinical settings, particularly when the purpose of screening and the availability of support are unclear [19, 34].Studies of social risk screening indicate that patients’ willingness to engage is shaped not only by the questions asked but by their expectations regarding what will happen after disclosure, including whether help will be offered and how information will be documented [19]. Loneliness is not the only sensitive or potentially stigmatized subject addressed in clinical care. Clinicians routinely ask about sexual health, substance use, interpersonal violence, mood, and other private experiences when the information is relevant to care. The ethical concern is therefore not that loneliness is uniquely inappropriate to discuss. Rather, systematic loneliness screening may be ethically distinctive because a positive response identifies a subjective and relational experience for which the meaning, preferred response, and appropriate locus of intervention may vary considerably. Disclosure may also create a reasonable expectation that the healthcare system can offer acknowledgment, further assessment, or support. When the purpose of screening, use of the information, and limits of available assistance are not made clear, routinization may weaken meaningful voluntariness even though patients technically retain the option not to answer.

Screening without adequate follow-up capacity can therefore undermine autonomy by eliciting sensitive information without providing meaningful choice or control over outcomes. When patients disclose loneliness but encounter limited or no response, screening may create emotional distress, disappointment, or a sense of abandonment, raising concerns related to nonmaleficence [21]. These harms are particularly salient when screening is embedded into routine workflows and framed as standard care, which may limit patients’ perception that participation is optional or that declining to disclose is acceptable [20]. Further, autonomy extends beyond obtaining responses to screening questions. Patients should also participate in decisions regarding whether screening occurs, how results are documented, whether referrals are desired, and which forms of support best align with their preferences, goals, and circumstances. Shared decision-making may therefore represent an important ethical safeguard when implementing loneliness screening.

Beneficence is also implicated at the patient level. While loneliness screening is often justified by its potential to identify individuals who could benefit from intervention, the absence of effective or accessible response options weakens this justification. Moreover, brief loneliness screening instruments necessarily simplify a complex, multidimensional experience that is shaped by interpersonal relationships, developmental history, culture, life circumstances, and individual perceptions of belonging [1, 2]. Although brief measures such as the UCLA-3 have demonstrated acceptable psychometric performance for population screening [35], they cannot fully capture the context, meaning, or causes of an individual’s loneliness. Consequently, identification alone does not ensure that clinicians understand the underlying drivers of a patient’s experience or that a standardized referral will be appropriate or beneficial. Without opportunities for contextual assessment and person-centered discussion, screening may risk reducing a complex human experience to a checklist item rather than facilitating meaningful support [1, 11].

Conversely and importantly, there are potential benefits of inquiry that are not limited to referral or measurable reduction in loneliness. Patients may benefit from having social health recognized as relevant to care, from receiving empathic acknowledgment, and from learning that loneliness is a legitimate subject for clinical discussion. A brief conversation may validate distress, reduce shame, or strengthen the therapeutic relationship even when a formal service is unavailable. These relational benefits should be included in any assessment of beneficence. At the same time, their possibility does not by itself justify indiscriminate or poorly implemented screening. The ethical value of asking depends on the manner of inquiry, the patient’s preferences, the clinician’s response, and the accuracy of expectations regarding available assistance.

### Provider-level ethical challenges

Loneliness screening without response capacity also generates ethical challenges for clinicians, particularly in relation to beneficence and professional responsibility. Studies document that clinicians often experience discomfort initiating conversations about loneliness and frustration when screening reveals needs they cannot address [25, 34]. This discomfort is compounded by limited training, uncertainty about appropriate next steps, and lack of familiarity with community-based resources [25, 36]. However, clinician discomfort alone does not establish that loneliness screening is unethical and should not be used to avoid clinically relevant conversations. The more significant concern arises when institutions require clinicians to identify loneliness without providing sufficient time, training, interpretive guidance, or pathways for proportionate response. Under those circumstances, clinicians may experience conflict between professional commitments to respond to disclosed suffering and organizational constraints that prevent meaningful action. This is best understood as a problem of program design and professional responsibility rather than as an independent objection to screening.

These experiences can create moral tension for clinicians, who may recognize patients’ needs but lack the tools, time, or institutional support to respond effectively. While the literature does not always frame this tension explicitly as moral distress, it reflects a misalignment between clinicians’ ethical commitments to beneficence and the constraints imposed by practice environments. When screening expands without parallel investment in response infrastructure, clinicians may be placed in ethically untenable positions where they are expected to identify vulnerability without being able to alleviate it.

### System-level and structural contributors

At the system level, ethical concerns related to justice and fairness become particularly salient. Loneliness and related social risks are not evenly distributed across populations; they are shaped by broader social and economic inequalities, including poverty, social exclusion, and structural disadvantage [23]. Clinicians serving high-need populations may therefore identify greater levels of loneliness while simultaneously facing the greatest resource constraints, creating a risk that screening amplifies rather than mitigates inequities. It should be noted that screening does not create the underlying social and structural inequalities associated with loneliness, nor does recognition itself make a patient more socially disadvantaged. The justice concern arises at the level of the screening program’s downstream distribution of benefits and burdens. When patients in different settings are equally likely to be asked about loneliness but have markedly different access to follow-up assessment, care coordination, or community resources, the benefits of identification may accrue disproportionately to those already receiving care in better-resourced systems. Patients in under-resourced settings may experience the burdens of disclosure and documentation without comparable opportunities for assistance. The relevant ethical question is therefore whether the screening pathway provides fair access to potential benefit, not whether screening caused the preexisting inequality.

### Justice, equity, and structural considerations

Justice-based ethical concerns are central to evaluating loneliness screening because both loneliness itself and the capacity to address it are unevenly distributed across populations and health systems. As a result, primary care practices serving populations with greater structural disadvantage may identify higher levels of loneliness while simultaneously facing greater constraints in staffing, time, and access to community-based resources [34]. This mismatch creates the ethical risk that loneliness screening produces unequal burdens and unequal benefits. Patients in under-resourced settings may be more likely to be identified as lonely but less likely to receive meaningful follow-up or support, while patients in better-resourced settings may experience screening as beneficial due to stronger referral networks and social infrastructure [20]. Empirical studies of social needs screening demonstrate that identification alone does not guarantee assistance and that referral success varies widely depending on local context [19, 25]. Identification of loneliness in populations already experiencing disadvantage is not inherently harmful and may generate clinically and socially valuable information. Recognition can support validation, epidemiologic understanding, resource planning, and advocacy. The justice concern arises when a health system introduces screening as a beneficial service but does not examine whether patients across sites and populations have comparable opportunities to receive any ensuing benefit. Ethical evaluation should therefore assess not only who is screened and who screens positive, but also who receives further assessment, who is offered support, who accepts it, and who ultimately gains from the program.

### Systematic screening, targeted assessment, and clinical identification

The terms *screening* and *identification* should not be treated as interchangeable. Systematic screening generally refers to the routine application of a standardized question or instrument to a defined population, including individuals in whom the concern has not otherwise been recognized. Such a program entails responsibilities extending beyond administration of the measure, including clear objectives, appropriate interpretation, documentation practices, follow-up procedures, quality assurance, and evaluation of equitable access to any resulting support.

By contrast, targeted assessment occurs when clinical history, life circumstances, observed distress, or known risk factors prompt further inquiry. Loneliness may also be identified through an open-ended conversation without use of a formal instrument. These approaches may permit more contextual and person-centered exploration and may be appropriate where formal screening infrastructure is limited. However, reliance solely on clinician judgment may produce inconsistent inquiry and may reproduce implicit biases regarding who is perceived as lonely or socially vulnerable. The morally relevant question is therefore not simply whether loneliness is asked about, but whether the method of identification, expectations created, and available responses are proportionate, transparent, and equitable.

### Structural competency and loneliness

The concept of structural competency provides a useful lens for understanding why loneliness screening raises justice concerns beyond individual clinician behavior or patient choice. Structural competency emphasizes that health outcomes and clinical encounters are shaped by upstream social, economic, and political forces, rather than solely by individual pathology or behavior [37]. From this perspective, loneliness is not merely an individual deficit, but a relational experience embedded within broader structural conditions, including labor markets, housing policies, social safety nets, and community cohesion.

### Ethical pathways forward: alternatives and mitigations

An ethically defensible approach to loneliness screening requires conditions that align identification with response. Screening should be contingent on the availability of referral pathways, clinician training, and community partnerships sufficient to offer meaningful support [24, 38]. Ethical screening also requires transparency with patients regarding the purpose of screening, the voluntary nature of disclosure, and the limits of available assistance [21]. Embedding loneliness screening within broader care models, rather than treating it as a standalone metric, may mitigate ethical risks. Reviews emphasize the importance of integrating screening with care coordination, social prescribing, and community engagement to ensure that identification leads to action [11, 24]. Such integration requires investment not only in clinical workflows but also in community infrastructure and cross-sector partnerships that can provide sustainable support for individuals experiencing loneliness.

### Alternative approaches to routine screening

Alternative approaches to universal screening may include targeted identification based on clinical judgment, stepped assessment strategies, or prioritizing intervention development before widespread screening. Such approaches may reduce the ethical risks associated with over-identification in settings lacking response capacity [20]. Analogous literature on behavioral health screening suggests that phased implementation and attention to follow-up infrastructure can improve ethical alignment between screening and care [39]. Additionally, clinicians might employ conversational approaches to assessing loneliness that are responsive to individual context and readiness, rather than relying solely on standardized brief tools. Such approaches allow for clinical discretion and can be tailored to settings where formal referral pathways are limited but where empathic acknowledgment and brief counseling may still offer benefit. The trade-off, however, is reduced standardization and potential inconsistency in who is asked and how concerns are documented.

### Implications for future research

Future research should examine ethical outcomes alongside clinical and implementation outcomes, including patient distress, clinician burden, and equity effects [20, 25]. Incorporating ethical considerations into study design and evaluation will strengthen the translational relevance of loneliness research and inform more responsible implementation strategies. Specifically, studies should assess:

1. Patient-reported experiences of screening, including acceptability, distress, perceived benefit, and satisfaction with follow-up;
2. Clinician experiences, including confidence, preparedness, moral distress, and perceived adequacy of available resources;
3. System-level outcomes, including referral completion rates, intervention uptake, and variation in outcomes across practice settings with different resource levels;
4. Equity impacts, including whether screening and intervention access differ by race, ethnicity, socioeconomic status, insurance type, or geographic location.

Such research would provide empirical evidence to inform ethical guidelines and policy decisions regarding the appropriate scope and conditions for loneliness screening in primary care.

### Professional and policy implications

At the professional and policy levels, ethical guidance is needed to ensure that loneliness screening aligns with available resources and does not become a symbolic or performative intervention. Policies that incentivize screening should be paired with investment in training, community resources, and infrastructure to support response [21, 23]. Ethical stewardship requires that health systems balance aspiration with accountability. Professional organizations can play a role in developing practice guidelines that specify the conditions under which loneliness screening is appropriate, including minimum standards for clinician training, referral pathway availability, and patient communication. Such guidelines should be informed by both empirical evidence and ethical analysis, and should be revisable as new evidence emerges.

Finally, policy efforts to address loneliness should extend beyond clinical screening to include investments in community infrastructure, social programs, and policies that address structural determinants of social connection. Loneliness is, fundamentally, a social and relational phenomenon, and addressing it effectively requires interventions that extend well beyond the clinical encounter. Health care can play a role in identifying and supporting individuals experiencing loneliness, but it cannot, and should not, be expected to solve a problem rooted in broader social conditions without corresponding societal investment.

## Conclusion

Loneliness screening in primary care reflects growing recognition of social connection as a determinant of health. However, ethical analysis reveals significant risks when screening outpaces intervention capacity. The ethical challenges identified in this analysis are not insurmountable, but they require careful attention to implementation context, system readiness, and equity considerations. Ethically defensible loneliness screening requires alignment between recognition, capacity, and accountability. This includes ensuring that adequate referral pathways, clinician training, and community partnerships are in place; that patients are informed about the purpose and limits of screening; and that screening initiatives are evaluated for their impacts on patient experience, clinician burden, and equity across diverse populations and practice settings.

## Data Availability

No datasets were generated or analysed during the current study.
